# Biological Assessment of Laser-Synthesized Silicon Nanoparticles Effect in Two-Photon Photodynamic Therapy on Breast Cancer MCF-7 Cells

**DOI:** 10.3390/nano10081462

**Published:** 2020-07-26

**Authors:** Ahmed Al-Kattan, Lamiaa M. A. Ali, Morgane Daurat, Elodie Mattana, Magali Gary-Bobo

**Affiliations:** 1 Aix Marseille University, CNRS, LP3 UMR 7341, Campus de Luminy, Case 917, 13288 Marseille, France; 2 IBMM, Univ Montpellier, CNRS, ENSCM, 34093 Montpellier, France; lamiaa.ali@umontpellier.fr (L.M.A.A.); emattana.ibmm@gmail.com (E.M.); magali.gary-bobo@inserm.fr (M.G.-B.); 3 Department of Biochemistry, Medical Research Institute, University of Alexandria, Alexandria 21561, Egypt; 4 NanoMedSyn, 15 avenue Charles Flahault, 34093 Montpellier, France; morgane.daurat@umontpellier.fr

**Keywords:** pulsed laser process, silicon nanoparticles, two-photon excited photodynamic therapy (TPE-PDT)

## Abstract

Driven by their distinctive physiological activities, biological properties and unique theranostic modalities, silicon nanoparticles (SiNPs) are one of the promising materials for the development of novel multifunctional nanoplatforms for biomedical applications. In this work, we assessed the possibility to use laser-synthesized Si NPs as photosensitizers in two-photon excited photodynamic therapy (TPE-PDT) modality. Herein, we used an easy strategy to synthesize ultraclean and monodispersed SiNPs using laser ablation and fragmentation sequences of silicon wafer in aqueous solution, which prevent any specific purification step. Structural analysis revealed the spherical shape of the nanoparticles with a narrow size distribution centered at the mean size diameter of 62 nm ± 0.42 nm, while the negative surface charge of −40 ± 0.3 mV ensured a great stability without sedimentation over a long period of time. In vitro studies on human cancer cell lines (breast and liver) and healthy cells revealed their low cytotoxicity without any light stimulus and their therapeutic potential under TPE-PDT mode at 900 nm with a promising cell death of 45% in case of MCF-7 breast cancer cells, as a consequence of intracellular reactive oxygen species release. Their luminescence emission inside the cells was clearly observed at UV-Vis region. Compared to Si nanoparticles synthesized via chemical routes, which are often linked to additional modules with photochemical and photobiological properties to boost photodynamic effect, laser-synthesized SiNPs exhibit promising intrinsic therapeutic and imaging properties to develop advanced strategy in nanomedicine field.

## 1. Introduction

Because of its fundamental specificity and selectivity, photodynamic therapy (PDT) is intensively explored in cancer therapy and even in various non-malignant diseases including infections [1,2]. In fact, PDT is a non-invasive therapeutic technique which can be combined with other therapeutic modalities (e.g., radiotherapy and chemotherapy) and it offers the possibility to use many photosensitizing agents, such as nanoparticles (NPs), in order to generate a maximum reactive oxygen species to induce cell destruction [3,4,5,6]. PDT can be exploited under two-photon excitation (TPE-PDT) in the near-infrared (NIR) wavelength region offering the possibility to work in deeper tissue (down to 2 cm). Moreover, thanks to the non-linear absorption, the PDT damage can be dramatically limited to the tumor area with a high spatiotemporal resolution [7,8,9].

Among popular theranostic tools used in TPE-PDT, such as organic molecules and quantum dots [10,11,12], nanoengineered particles based on silicon and their derivative formulations have gained significant interest. Indeed, silicon element is one of the most abundant mineral on earth with distinctive physiological activities such as bone mineralization and transduction mechanism [13,14,15,16,17]. Beside their relevant antibacterial properties, silicon nanoparticles (SiNPs) can be biodegraded into orthosilicic acid Si(OH)_4_ without specific induced toxicity [16]. In addition, the Si chemistry exhibits attractive surface reactivity toward functional molecules such as proteins, drugs, target agents and biological receptors. Finally, SiNPs offer also the possibility of advanced theranostic modalities, such as photoluminescence-based imaging, photodynamic and hyperthermia therapies [18,19,20,21,22]. Mostly NPs based on silicon, exploited in TPE-PDT are synthesized through chemical routes such as sol-gel condensation/polymerization of organosilane precursor, leading to the formation of mesoporous-silica NPs with the necessity to employ several stages of switching solvents and purification steps before use in biological medium [3,4]. Anodical electrochemical etching method is another process reported in literature to generate porous SiNPs in hazardous hydrofluoric acid (HF) solvent from bulk silicon [23,24]. Beside the risks of residual contamination, this process also requires mechanical scratching step leading to the production of various size and shape nanoparticles, which is less consistent with targeted biomedical applications.

In this context, Pulsed Laser Ablation in Liquid (PLAL) appears as relevant process to generate ultraclean NPs [25,26,27,28]. In fact, such process is based on the interaction of an ultrafast femtosecond (fs) pulse laser beam with a solid target (bulk material or water dispersed powder) in aqueous solution, leading naturally to the generation of stable “bare” colloidal NPs suspension free from any ligands, dispersant agents and other by-product synthesis. Moreover, by playing on numerous laser parameters (fluence, frequency, laser beam duration, laser-ablative geometry, etc.) and physicochemical parameters, including solvent properties (aqueous/organic, pH, ionic forces, etc.), it is possible to monitor several structural NPs properties such as size distribution, oxidation rate, and even to proceed to “in situ” (during laser process) functionalization with biomolecules [25,28].

We have thus recently shown the possibility to fabricate biocompatible and biodegradable SiNPs with manageable size distribution and dissolution rate properties [28]. Additionally, the in vivo tests conducted at single intravenous injection dose of 20 mg mL^−1^ on nude mice model combined with a panel of biochemical parameters and other microscopic and histological studies confirmed the complete safety properties of SiNPs. The therapeutic functionality was proved thanks to intra-tumor radiofrequency (RF)-induced hyperthermia tests conducted on Lewis lung carcinoma with efficient tumor inhibition even at relative low concentration [22,29]. Recently, laser-synthesized NPs were also tested as functional modules (additives) in innovative scaffold platforms for tissue engineering applications [30,31,32].

Despite the promising results obtained, the theranostic capability of laser-synthesized SiNPs still under explored, especially in TPE-PDT treatment modality, where little information is available. In this work, monodispersed bare SiNPs were elaborated thanks to facile protocol based on laser ablation and fragmentation sequences without specific purification step. Moreover, we assess the in vitro cytotoxic effect of SiNPs on human breast adenocarcinoma cell line (MCF-7), the cellular uptake and their luminescence emission using confocal microscopy at NIR region. In addition, the photodynamic effect under two photon excitation configuration, and reactive oxygen species production were described, on MCF-7 cells first and on other cancer and healthy cells to determine the biomedical application of such a strategy.

The activation of SiNPs by two-photon excitation offers several advantages over classical PDT, mainly in thick tissues. Indeed, this allows an increased penetration depth of the excitation beam thanks to the use of near infrared (NIR) wavelengths, and an intrinsic three-dimensional resolution allowing a better spatial control of singlet oxygen production [33]. In addition, to achieve efficient treatments, the SiNPs should exhibit a large two-photon absorption (2PA) cross-sections in the biological window (700–1000 nm) and high singlet oxygen production quantum yields [34]. In the presented work, we have demonstrated that Si NPs could be used for two-photon excitation imaging or PDT, even in thick tissues.

## 2. Materials and Methods

### 2.1. Laser-Synthesis of Colloidal Si Nanoparticles

Colloidal suspension of SiNPs was prepared using Yb:KGW femtosecond (fs) laser radiation (Amplitude Systems, Bordeaux, France, 1025 nm, 480 fs, 1 kHz), employed in ablative configuration. Basically, a silicon wafer target (1 cm × 1 cm) was placed into a 7 mL ultrapure water solution and ablated for 20 min under ambient atmosphere conditions. The laser beam was focused on the surface target with a help of 75 mm focal lens. To promote an optimal surface ablation, the target was constantly moved at the speed of 2 mm s^−1^ with a motorized linear translational stage (Newport). The Si NPs colloidal solution was then collected and treated for a second time under fs laser in fragmentation configuration for 3 min to ensure a homogenous Si NPs size distribution. The solution was continuously stirred during the treatment to ensure a uniform fragmentation.

### 2.2. Physicochemical Characterizations of SiNPs and Preparation for Biological In Vitro Studies

A high-resolution transmission of electronic microscopy (HR-TEM) (JEOL JEM 3010, Tokyo, Japan) operating at 300 kV and coupled with X-ray diffraction mode was employed to perform structural characterizations. Samples were prepared by placing a drop of Si NPs solution on copper TEM grids (200 mesh) (Oxford instruments, Abingdon, UK) coated with carbon support film and allowing it to dry before TEM analyses. ζ-potential measurements, using a Zetasizer Nano ZS (Malvern Panalytical, Malvern, UK), were also performed to evaluate the surface charge of SiNPs. Extinction spectra of SiNPs solutions were measured by a UV–VIS spectrophotometer (UV-2600, Shimadzu, Tokyo, Japan) using 10 mm optical path length quartz cuvettes. To proceed to biological in vitro studies, a calibrated SiNPs solution at the concentration of 8.7 mg mL^−1^ was prepared based on centrifugation step. The concentration was measured by an induced coupled plasma mass spectroscopy (ICP-MS) and statistic size distribution was performed on more than 300 SiNPs using ImageJ software (National Institutes of Health (NIH), Bethesda, MA, USA).

### 2.3. Cell Culture

All cell lines were purchased from American Type Culture Collection (ATCC). Human breast adenocarcinoma cell line (MCF-7) was maintained in Dulbecco’s Modified Eagle’s Medium/F12 (DMEM/F12) supplemented with 10% fetal bovine serum (FBS) and 1% penicillin/streptomycin (P/S). Human breast adenocarcinoma cell line (MDA-MB-231) were maintained in DMEM supplemented with 10% FBS and 1% P/S. Healthy fibroblasts (FS 20-69) and human hepatocyte carcinoma cell line (Hep-G2) was cultured in RPMI medium supplemented with 10% FBS and 1% P/S. Cells were allowed to grow in humidified atmosphere at 37 °C under 5% CO_2_.

### 2.4. In Vitro Toxicity Study

MCF-7 cells were seeded in 96-well plate. After 24 h of cell growth, cells were treated with increasing concentrations of SiNPs (from 1.25 to 200 µg mL^−1^) for 72 h. Control cells were treated with vehicle. Toxicity was evaluated using the colorimetric assay (4,5-dimethylthiazol-2-yl)-2,5-diphenyltetrazolium bromide (MTT) of living cells. Briefly, cells were incubated for 4 h with 0.5 mg mL^−1^ of MTT in media. The MTT/media solution was then removed and the precipitated crystals were dissolved in equal volume solution of ethanol/DMSO. The solution absorbance was read at 540 nm. The optical density (OD) values are directly correlated with the number of living cells in well.

### 2.5. Cellular Uptake

The cellular uptake of SiNPs was performed using confocal fluorescence microscopy on living cells. Cells were plated on Lab-Tek II Chambered Coverglass (Nalge Nunc International Ref 155382) in 0.5 mL culture medium for 24 h. Then, cells were treated with 50 µg mL^−1^ of SiNPs for 24 h. Control cells were treated with vehicle. Fifteen minutes before the end of incubation, cells were stained with CellMask^TM^ Orange plasma membrane stain (Invitrogen, Cergy Pontoise, France, C10045) for membrane staining at a final concentration of 5 μg mL^−1^. Then cells were washed two times with culture medium. Confocal fluorescence microscopy was performed on living cells at a 900 nm excitation wavelength for SiNPs and 561 nm for cell membranes.

### 2.6. Two-Photon Excitation Photodynamic Therapy

Healthy and cancer cells were seeded in 384-multiwell glass-bottom plate (thickness 0.17 mm) with a black polystyrene frame. Cells were allowed to grow for 24 h, and then treated with or without 50 µg mL^−1^ of silicon nanoparticles for 24 h. After the incubation time, cells were exposed or not to laser beam at 900 nm using Carl Zeiss confocal microscope LSM 780 (maximum laser power input = 3 W). The laser beam was focused by a microscope objective lens (Carl Zeiss 10×/0.3 EC Plan-Neofluar). Four different areas of the well, their total area is equivalent to the half area of the well, were irradiated by 3 scans of 1.57 s duration. The scan size does not allow irradiating more areas without overlapping. Two days after irradiation, the phototoxicity effect of SiNPs was assessed using MTT assay as previously described and values were corrected according to the following formula: Abs control – 2 × (Abs control − Abs SiNPs). Statistical analysis was carried out using student’s *t*-test.

### 2.7. Reactive Oxygen Species (ROS) Detection

MCF-7 cells were seeded in 96-well plate glass bottom half-area, and then cells were treated with or not 50 µg mL^−1^ of SiNPs for 24 h. The detection of intracellular ROS was realized using DCFDA Cellular ROS Detection Assay Kit (abcam). Briefly, cells were incubated with or without 20 µM of 2′, 7′-dichlorofluorescein diacetate (DCFDA) for 45 min at 37 °C. Then, cells were irradiated or not using two-photon excitation microscope (3 scans × 1.57 s) at 900 nm. After irradiation, cells were washed twice then exposed to imaging using Carl Zeiss confocal microscopy at 488 nm, objective 20×. In the presence of ROS, as a consequence of PDT, the non-fluorescent DCFDA is oxidized into the green fluorescent dichlorofluorescein (DCF).

### 2.8. PDT on 3D Spheroids from MCF-7 Cells

3D MCF-7 spheroids were prepared by hanging drop method. In brief, drops of 30 µL of cell suspension containing 500 cells were added into the lid of 60 mm × 15 mm tissue culture dish, then the lid was inverted onto bottom chamber containing PBS supplemented with 20% FBS. Spheroids were incubated for 5 days. After incubation periods (5 or 10 days), spheroids were transferred into Lab-Tek II Chambered Coverglass (Nalge Nunc International Ref 155382) and treated with 50 µg mL^−1^ of SiNPs for 24 h. Then, spheroids were imaged, irradiated or not using two-photon excitation microscope (3 scans × 1.57 s) at 900 nm. Spheroids were imaged 48 h after irradiation.

## 3. Results and Discussions

**Figure 1:**
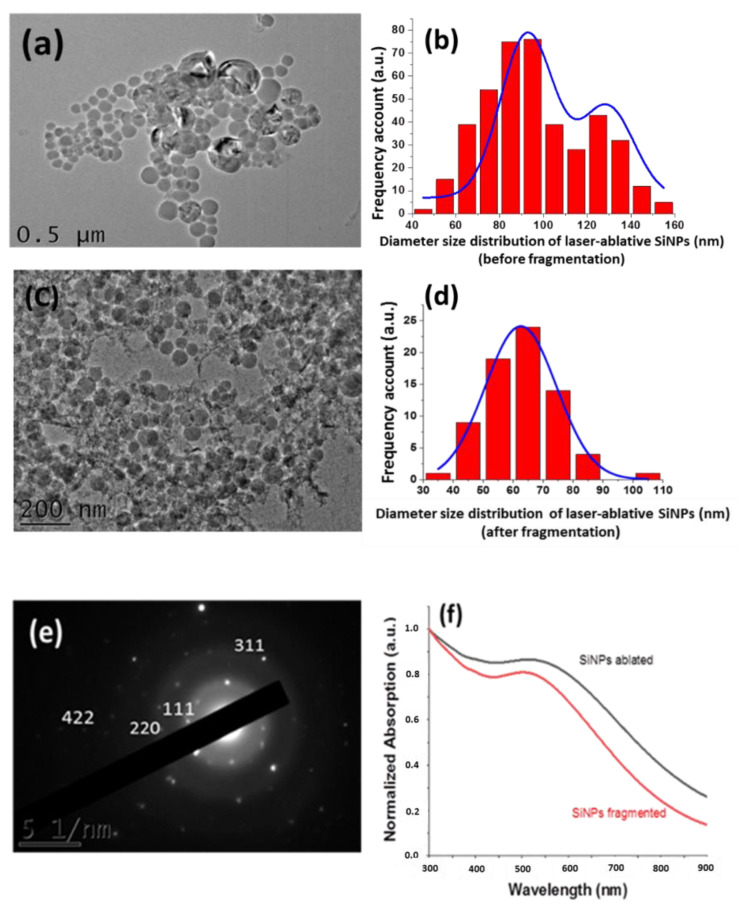
Typical HR-TEM image of SiNPs produced by laser-synthesis before (**a**) and after (**c**) fragmentation with corresponding diameter size distribution (**b**,**d**) respectively. (**e**)Typical X-ray diffraction pattern of SiNPs obtained after fragmentation. (**f**) Absorption spectra of SiNPs elaborated by laser-synthesized process after ablation or fragmentation step in pure water.

To elaborate SiNPs, we used a facile protocol based on the combination of laser ablation and fragmentation methods starting from silicon wafer immersed in ultrapure water at ambient atmosphere conditions [31]. In comparison to our previous work where the SiNPs were generated from water dispersed silicon micro-powder particles [28], this approach has the advantage to generate immediately SiNPs in the supernatant solution separated from the wafer target, which consequently limits any specific post-recovery stage and prevents purification step from unreacted material. Structural characterization of SiNPs was carried out after ablation and fragmentation steps, using HR-TEM coupled with X-ray diffraction analysis as shown in Figure 1.

After ablation, SiNPs appeared with typical spherical laser-ablated NPs with bimodal size distribution centered at 92 ± 5.76 nm and 129 ± 10.50 nm as it was determined by statistic measurement (Figure 1a,b). The presence of bimodal size dispersion of SiNPs can be explained by two competing mechanisms: (a) direct radiative ablation and (b) secondary ablation due to a collapse of cavitation bubble leading to a relative broad size distribution of SiNPs [31,35,36,37].

To reduce the mean size of SiNPs and to ensure a monodispersed size distribution, the colloidal SiNPs solution was then treated under fragmentation step for 3 min. In fact, as we reminded in our previous work, femtosecond laser fragmentation geometry has the advantage to generate weaker cavitation phenomena and white light supercontinuum in liquid leading to uniform fragmentation process [35,36,37]. The fragmented SiNPs appeared thus spherical (Figure 1c), with a narrow size distribution centered at 62 ± 0.42 nm (Figure 1d). Thanks to X-ray diffraction measurements, all SiNPs prepared by femtosecond laser methods exhibited characteristic atomic plans orientation of Si associated with crystalline state (Figure 1e). Moreover, ζ-potential analysis (Zetasizer Nano ZS, Malvern, UK) revealed that the NPs surface charge exhibits a negative value of −40 ± 0.3 mV due to the partial oxidation occurring on the NPs surface, as we have previously reported [28]. This leads to the formation of oxidized species (e.g., SiO*_x_* 0 ≤ *x* ≤ 2), which their charge ensures a great stability for several months by electrostatic repulsion between SiNPs. Furthermore, the SiO*_x_* chemistry makes the NPs surface very reactive toward additional functionalization [28]. Moreover, a calibrated SiNPs solution was then prepared at a concentration of 8.7 mg mL^−1^ measured by ICP-MS technic before proceeding to biological studies. Finally, optical property of SiNPs before and after fragmentation was also characterized in water using UV-visible absorption measurements as shown in Figure 1f. All absorption spectra appeared characteristic to SiNPs with a broad absorbance in the region of 300 nm to 800 nm with a shoulder peak in the region of 500 nm. Moreover, a slight shift of the shoulder peak after fragmentation step to blue-green region was observable which is attributed to the change of SiNPs size and effect of quantum confinement [38,39].

**Figure 2:**
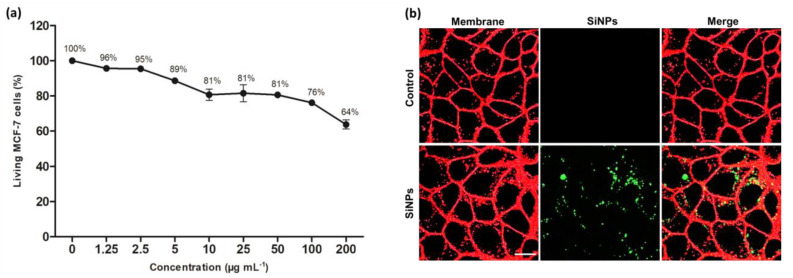
(**a**) Cell viability (%) of MCF-7 cells treated with increasing concentrations of SiNPs (from 1.25 to 200 µg mL^−1^) for 72 h. Data are presented as (mean ± SEM) of two independent experiments realized in triplicate; (**b**) Confocal microscopy imaging of living MCF-7 breast cancer cells after 24 h of incubation with or without (Control) 50 µg mL^−1^ of SiNPs and excited at 900 nm. Scale bar 5 µm.

The SiNPs toxicity on MCF-7 cells was assessed from the concentration 1.25 µg mL^−1^ to 200 µg mL^−1^ using MTT assay, a commonly used colorimetric assay in which the produced color is directly proportional to the number of metabolically active viable cells [40]. As shown in Figure 2a, toxicity was relevant but low, up to 100 µg mL^−1^ of SiNPs. For instance, at 50 µg mL^−1^ and 100 µg mL^−1^, cell viability values were 81 ± 1% and 76 ± 2%, respectively. It was observed that the cell viability reached 64 ± 4% at 200 µg mL^−1^ of SiNPs, which is still quite acceptable. According to these results, the concentration 50 µg mL^−1^ has been chosen for the following experiments. The imaging potential and the uptake of SiNPs by MCF-7 cells were investigated using confocal fluorescence microscopy at 900 nm as shown in Figure 2b, which demonstrated that SiNPs were endocytosed by MCF-7 cancer cells and emitted a luminescence when excited at 900 nm with a pulsed laser.

**Figure 3:**
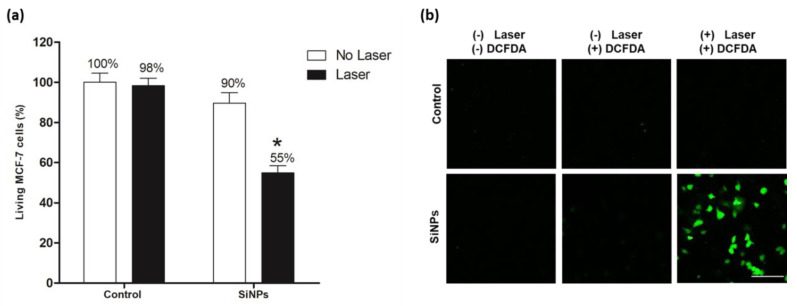
(**a**) TPE-PDT effect of SiNPs incubated 24 h with MCF-7 at 50 µg mL^−1^. Cells were irradiated with pulsed laser at 900 nm (3 scans × 1.57 s). Data are presented as (mean ± SEM) of two independent experiments realized in triplicate. * Statistical significance (*p* < 0.05) for silicon nanoparticles laser versus control no laser (student’s *t*-test); (**b**) Detection of intracellular reactive oxygen production by DCFDA in MCF-7 cells treated with 50 µg mL^−1^ of SiNPs and irradiated at 900 nm (3 scans × 1.57 s). Green fluorescence indicates ROS production as a consequence of PDT effect. Scale bar 30 µm.

Because of two-photon irradiation in the NIR area is preferred due to its deeper tissue penetration and safety, the photodynamic efficiency of SiNPs using TPE was evaluated thanks to Carl Zeiss confocal microscope LSM 780 (maximum laser power input = 3W). As shown in Figure 3a, no significant cell death was detected in control cells exposed to irradiation at 900 nm (3 scans × 1.57 s) in comparison to non-irradiated cells, which indicated the safety of our experimental parameters. When MCF-7 cells were exposed to 50 µg mL^−1^ SiNPs during 24 h, in absence of laser exposition, a slight but not significant cell death about 10% was detected. In contrast, MCF-7 incubated with 50 µg mL^−1^ SiNPs for 24 h and exposed to irradiation, exhibited a significant cell death of 45%. This cell death suggested strongly a PDT efficiency of SiNPs under 900 nm excitation wavelengths. Detection of intracellular ROS as a consequence of PDT effect was evaluated using a DCFDA kit. Results showed a green fluorescence signal in cells treated with SiNPs and exposed to irradiation by pulsed laser (Figure 3b). In contrast, no green fluorescence was detected in controls under the same experimental conditions. These results demonstrate the production of ROS by SiNPs under TPE.

**Figure 4:**
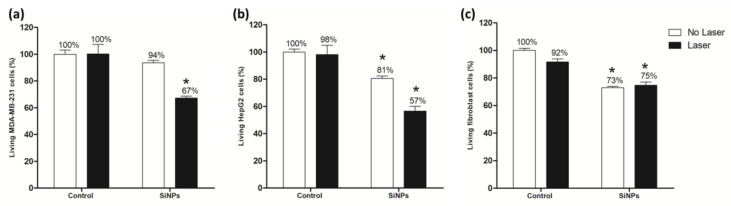
Two-photon excitation PDT effect of SiNPs on MDA-MB-231 cells (**a**), HepG2 cells (**b**) and fibroblast cells (**c**) treated with 50 µg mL^−1^ of NPs for 24 h. Cells were irradiated with pulsed laser at 900 nm (3 scans × 1.57 s). Data are presented as (mean ± SEM) of two independent experiments realized in triplicate. * Statistical significance (*p* < 0.05) for SiNPs laser versus control no laser (student’s *t*-test).

In order to prove the efficiency of these SiNPs for cancer cells therapy under pulsed laser excitation, we performed the same experiment on various human cell lines. First, a second breast cancer cell line was used (MDA-MB-231) and results demonstrated that without stimulus SiNPs induced no significant cell death; in contrast, under two-photon excitation, a decrease in living cells about 33% was measured (Figure 4a). Then a liver cancer cell line (HepG2) was also studied and data showed that SiNPs incubation induced 19% cell death and after pulsed laser stimulation, 43% cell death was quantified (Figure 4b). Finally, heathy cells (fibroblasts), already known to be less efficient to internalize nanoparticles, were incubated with SiNPs and irradiated (Figure 4c) [41]. Data demonstrated a decrease in living cells without light stimulus (27%), but interestingly, the laser excitation doesn’t induce any supplementary cell death (25%). This suggests an absence of PDT activity on these healthy cells, probably due to low nanoparticles internalization and consequently low singlet oxygen production inside the cells. This could confer specificity in the treatment of cancer without acting on healthy cells and thus limiting side effects.

**Figure 5:**
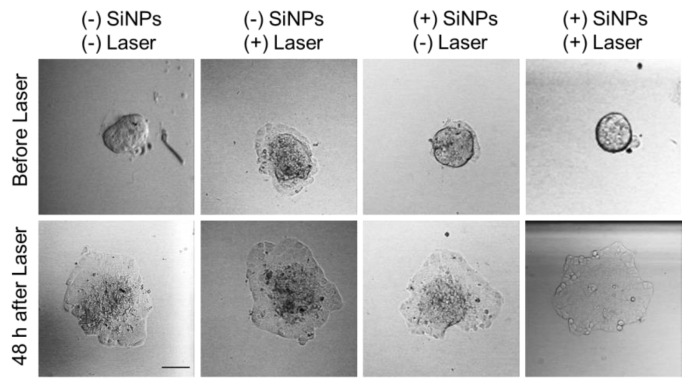
Two-photon excitation PDT effect of SiNPs on 3D cultures. Spheroids from MCF-7 cells were treated or not with 50 µg mL^−1^ of SiNPs for 24 h and irradiated or not with pulsed laser at 900 nm (3 scans × 1.57 s). Pictures are representative of the whole. Scale bar: 100 µm.

Finally, we decided to study the therapeutic potential on 3D culture. In fact, two-photon and near infra-red excitation are known to penetrate deeper in tissue than monophotonic and visible wavelength. We cultivate cellular bodies with spheroid structure, and separate them in function of their size. We investigated the effect of SiNPs treatment and two-photon excitation on “small spheroids” (around 1.5 × 10^4^ cells) and “big spheroids” (around 5 × 10^5^ cells). The data demonstrated that no effects were detected on big spheroids, which grew equally under treatment or in control conditions (data not shown). In contrast, on small spheroids, the incubation with 50 µg mL^−1^ SiNPs for 24 h followed by two-photon excitation (3 scans of 1.57 s at 900 nm) induced a modification in their structure (Figure 5). Indeed, in all control conditions (without any treatment or with SiNPs alone or with laser alone) the initial spheroids exhibited a well-defined 3D structure (Figure 5, Before Laser). Two days after, the 3D structure was always present and some cells spread around the spheroid generating a monolayer of cancer cells. In contrast, the treatment with SiNPs and laser induced a disappearance of the 3D structure, only a monolayer of cells was present (Figure 5, 48 h after Laser). This was generally observed in all the 3D cultures selected at the beginning of the experiment on the basis of their homogenous size, one representative picture of the majority was presented in Figure 5. These results are encouraging and suggest a possible using for tumor therapy.

These first results are a direct demonstration of the validity to use SiNPs synthesized by ultrafast fs laser process as promising tools for TPE-PDT gathering in a single nanoplatform the theranostic capability. In fact, our experiments were conducted on primitive SiNPs formulation in comparison to their chemically synthesized counterparts, which are often linked to additional modules to enhance PDT efficiency [8]. Moreover, very little information is available on the mechanism of singlet oxygen (^1^O_2_) generation from SiNPs prepared by fs laser process. Rioux and coworkers proposed that the ^1^O_2_ generation is due to energy transfer from exciton confined in Si nanocrystals to oxygen molecules as Kovalev’s model suggested for porous SiNPs prepared by electrochemical route [23]. Thus, several parameters such as surface chemistry, efficient absorption in NIR region, selectivity toward cancer cells, etc. have to be taken into account to optimize TPE-PDT efficiency of SiNPs and to clarify the mechanism of ^1^O_2_ generation.

## 4. Conclusions

In conclusion, thanks to facile laser-synthesis protocol, we demonstrated the possibility to obtain ultrapure SiNPs of 62 ± 0.42 nm with relevant imaging and PDT treatment modalities. The two-photon excited photodynamic therapy results were promising. Indeed, several cell lines were studied and the best result was obtained on MCF-7 cancer cells since 45% of death was obtained and the ROS production was demonstrated. In addition, the cellular uptake of SiNPs and their luminescence emission at visible wavelength was clearly observed. This research opens up exciting perspectives on the development of powerful photosensitizers based on laser-synthesized SiNPs, which are barely used in TPE-PDT treatment modality. Other parameters, such as physicochemical properties (surface chemistry, size, etc.) of SiNPs and selectivity toward cancer cells, have to be considered to clarify the mechanism of phototoxicity in view to generate a maximum TPE-PDT efficiency.
